# The Meaning of a Positive Living Environment in Nursing Homes: a Scoping Review and Concept analysis

**DOI:** 10.1093/geroni/igaf122.4028

**Published:** 2025-12-31

**Authors:** Lien Janssens, Lisa Geyskens, Ellen Vlaeyen, Bernadette Dierckx de Casterlé, Mieke Deschodt

**Affiliations:** KU Leuven, Balen, Vlaams-Brabant, Belgium; KU Leuven, Balen, Vlaams-Brabant, Belgium; KU Leuven, Balen, Vlaams-Brabant, Belgium; KU Leuven, Balen, Vlaams-Brabant, Belgium; KU Leuven, Balen, Vlaams-Brabant, Belgium

## Abstract

Many studies explore elements of nursing home living environments that contribute to concepts such as a sense of home, quality of life, or thriving. Yet, the overarching understanding of a ‘positive living environment’ remains unclear. We aimed to clarify what constitutes a positive nursing home environment and identify its key elements. We combined scoping review with concept analysis methodology to determine the core characteristics of such an environment (defining attributes), the required conditions (antecedents), and outcomes (consequences). Five databases were searched for original qualitative studies published in the past 20 years exploring resident, staff, and informal caregivers’ perspectives. In total, 146 studies met inclusion criteria and were categorized based on relevance. We analyzed the 19 highly relevant studies reflecting residents’ perspectives. Our findings show that a positive living environment is a flexible concept and is co-created through dynamic interaction between residents, staff, and the broader context. The environment is perceived as positive when residents can continue being themselves. This includes experiencing a balance between support and independence, building and maintaining relationships, engaging in meaningful activities, receiving quality care, staying connected with the outside community and being involved in nursing home life. Achieving this requires organizational conditions such as quality caregivers, a supportive physical environment, as well as resident-related factors including a positive mindset and acceptance. The consequences include dignity, belonging, peace of mind, feeling at home and overall well-being. These elements are closely interconnected. Further research should examine these interrelationships to identify changes most likely to improve the overall environment.

